# Probability distributions of helminth parasite burdens within the human host population following repeated rounds of mass drug administration and their impact on the transmission breakpoint

**DOI:** 10.1098/rsif.2021.0200

**Published:** 2021-04-28

**Authors:** Benjamin S. Collyer, Roy M. Anderson

**Affiliations:** ^1^ MRC Centre for Global Infectious Disease Analysis, School of Public Health, Imperial College London, London, UK; ^2^ London Centre for Neglected Tropical Disease Research, Department of Infectious Disease Epidemiology, Imperial College London, London, UK

**Keywords:** helminths, breakpoint, aggregation

## Abstract

The existence of multiple stable equilibria in models of parasitic helminth transmission was a ground-breaking discovery over 30 years ago. An implication of this discovery, that there is a level of infection below which transmission cannot self-sustain called the transmission breakpoint, has in part motivated the push towards the elimination of many human diseases caused by the multiple species of helminth worldwide. In the absence of vaccines, the predominant method in this push towards elimination is to repeatedly administer endemic populations with anthelmintic drugs, over several treatment rounds, in what has become to be known as mass drug administration (MDA). MDA will inevitably alter the distribution of parasite burdens among hosts from the baseline distribution, and significantly, the location of the transmission breakpoint is known to be dependent on the level of aggregation of this distribution—for a given mean worm burden, more highly aggregated distributions where fewer individuals harbour most of the burden, will have a lower transmission breakpoint. In this paper, we employ a probabilistic analysis of the changes to the distribution of burdens in a population undergoing MDA, and simple approximations, to determine how key aspects of the programmes (including compliance, drug efficacy and treatment coverage) affect the location of the transmission breakpoint. We find that individual compliance to treatment, which determines the number of times an individual participates in mass drug administration programmes, is key to the location of the breakpoint, indicating the vital importance to ensure that people are not routinely missed in these programmes.

## 1. Introduction

A defining feature of the epidemiology of macroparasites is that the parasite burden of an individual is positively correlated to infectiousness and morbidity [1]. These macroparasites are largely helminth species (digeneans and nematodes) some of which are widespread infections of humans and livestock and a major health burden in regions of endemic infection. The generation time of macroparasites in the human host is typically on the scale of years, and hosts who harbour large numbers of parasites are responsible for a much greater proportion of transmission than those who are infected with smaller burdens. A phenomenon that is typically observed when measuring the intensity of parasitic infections within a population is that the distribution of parasites among hosts is overdispersed where the variance in parasite load is much larger than the mean value. The negative binomial probability model is widely used to describe these distributions of parasite burdens. Stated more simply, there tends to be a small number of hosts who harbour very large burdens while the majority of hosts harbour low burdens. Typically, over 80% of the parasites are harboured by fewer than 20% of the hosts [2].

The negative binomial distribution is typically a good fit to observed patterns in both humans and other vertebrate host species. The distribution has two parameters, the mean, m, and an aggregation parameter, *k*, which is inversely related to the degree of over dispersion and hence is small in value when parasites are highly aggregated within the host population. The distribution converges to Poisson in the limit *k* → ∞ (the Poisson distribution is a close approximation for *k* > 5) and corresponds to parasites infecting each host independently with equal probability, while *k* being small indicates high aggregation. The negative binomial probability model is chosen largely for convenience as a flexible distribution which fits a large range of ecological data, and which is unspecific about possible causal mechanisms which generate it [2]. It can arise from compounding Poisson distributions with different mean values or by generalization [3].

Parasitic worms, also known as helminths, are a leading cause of human morbidity around the globe. Among the important macroparasites that infect humans, it is estimated that soil-transmitted helminths, schistosomes and filarial worms currently infect over one billion people worldwide, disproportionally affecting low-income and developing countries in sub-Saharan Africa, Asia and the Americas. Controlling the morbidity of these diseases is primarily performed by large-scale deworming programmes employing anthelmintic drugs, referred to as mass drug administration (MDA), in which drugs are supplied to whole communities at a given frequency (once a year or more or less frequently depending on the intensity of transmission as measured by the basic reproductive number *R*_0_) to suppress the prevalence and average intensity of infection. These interventions may have a major effect on the distribution of worm burden, and may, in some circumstances, invalidate the common assumption that the distribution is well characterized by a negative binomial distribution. An example of such a situation would be when individual compliance to treatment varies greatly in a population, such that a few individuals never receive treatment either via personal choice or poor access to treatment provision.

The importance of the degree of parasite aggregation to the transmission dynamics of these helminth parasites has been documented in a number of publications. Mathematical models of transmission suggest the existence of three possible equilibria in the mean parasite burden, where two stable states of endemic infection and parasite extinction are separated by an unstable state which is termed the transmission breakpoint [1,4,5]. The quantitative difference between the mean worm load at the unstable state and parasite extinction decreases as the degree of parasite aggregation rise and the two converge as *k* tends to zero [5,6].

Good quality data describing the distributional changes in parasite loads under repeated MDA rounds is scarce because it is not possible to directly count worm burdens in humans except via worm expulsion or at autopsy. Typically, diagnostics measure the intensity of infection using eggs counts from urine, stool or blood samples or DNA detection (qPCR) methods. However, information is beginning to emerge from large scale trials of how best to conduct deworming programmes, such as TUMIKIA and DeWorm3, that suggest under repeated rounds of treatment the degree of worm aggregation in the targeted communities rises steeply [7–9]. The generative mechanism or mechanisms are poorly understood at present, but non-compliance to treatment in a small proportion of the population looks to be one of great importance [10,11]. In this paper, we examine how this change in the degree of aggregation, shifting the pattern away from the negative binomial assumption impacts on the transmission dynamics if the parasite, and most importantly how it influences the existence of a transmission breakpoint. Employing a probabilistic model, we focus on the expected effect of MDA on a population of helminth parasites which prior to intervention is distributed as a negative binomial among its human host population.

We characterize MDA by the number of repeated rounds, the coverage (the proportion of people who receive treatment), the efficacy of the drug regimen and the degree of compliance as measured by the correlation of an individual's treatment over repeated rounds to record how past behaviour in treatment dictates future behaviour. By considering these four factors, we derive exact expressions for the parasite distribution following rounds of repeated MDA, and compare the resulting distributions to the negative binomial distributions with the same moments up to second order. We compare our analytic results, which ignore the transmission dynamics between MDA rounds, to those generated by a numerical experiment in which a stochastic transmission model simulates the dynamics between MDA rounds. We also generate analytical expressions for the mean and a measure of parasite aggregation (using various statistics such as *k* and the variance to mean ratio) as the system relaxes to equilibrium for a linear model, which allows us to account for the dynamics of parasite aggregation over time as influenced by many repeated MDA rounds. Finally, we use our derived values for the mean and aggregation after successive MDA rounds to determine if it is possible to reach the transmission breakpoint for different coverages and levels of non-compliance.

## 2. A simple probability model of repeated mass drug administration

### 2.1. The general post-mass drug administration distribution

We begin with an initial population of parasites that is distributed as a negative binomial with mean m_0_ and aggregation *k*_0_, and let *X*_0_ be a random variable sampled from this distribution. The probability generating function for this negative binomial distribution is the following expectation

2.1 $$\begin{array}{c} G_{X_{0}}(z)=E\;z^{X_{0}}={\left(1+\frac{m}{k}(1-z)\right)}^{-k}. \end{array}$$

We suppose that there are *n* rounds of chemotherapy, and that in each round a fraction of the population c∈(0,1)  is randomly selected for treatment. Chemotherapy is modelled by assuming the drug used has an efficacy, *ɛ* ∈ (0, 1), so that for each individual parasite within the host the probability that the drug kills the parasite is *ɛ*. For a single round of chemotherapy, this means that if an individual host has a random initial parasite burden *X*_0_, and this host is selected for the chemotherapy, then post chemotherapy the burden, *X*_1_ conditioned on X_0_, has distribution *X*_1_|*X*_0_ ∼ Binomial(*X*_0_, 1 − *ɛ*). The probability generating function for the binomial distribution is $G_{X_{1}|X_{0}=x}(z)={\left(\varepsilon +(1-\varepsilon )z\right)}^{x}$ so using the law of total expectation we find that the probability generating function for the post MDA distribution is

2.2 $$\begin{array}{rl} G_{X_{1}}(z) & =E_{X_{0}}\;G_{X_{1}|X_{0}}(z)=G_{X_{0}}(\varepsilon +(1-\varepsilon )z)\\ & ={\left(1+\frac{m}{k}(1-\varepsilon )(1-z)\right)}^{-k}, \end{array}$$

and hence the post-MDA distribution for those people who receive treatment in a single round is a negative binomial with mean *m*_0_(1 − *ɛ*) and aggregation *k*_0_. (This result or one very close using moments is in Anderson & May, 1991, and detailed in Anderson *et al.* 2016, based on compounding distributions with different means to represent either age, exposure to infection or acquired immunity [1,12].) Applying this reasoning inductively, and ignoring transmission dynamics between MDA rounds, we find that the distribution after *n* rounds of MDA is

2.3 $$\begin{array}{c} P(X_{\mathrm{post}}=i)=\overset{n}{\underset{\;j=1}{\sum }}\pi_{j}P(X=i|N=j) \end{array},$$

where *π_j_* is the probability of an individual attending j out of the n rounds, and *X*|*N* = *j* ∼ NegBin(*m*_0_(1−*ɛ*) ^*j*^, *k*_0_). If we denote *N* as a random variable sampled from the distribution π, then the probability generating function of the distribution (2.3) can be written as

2.4 $$\begin{array}{c} G_{X_{\mathrm{post}}}(z)=E_{N}\left[{\left(1+\frac{m_{0}}{k_{0}}{\left(1-\varepsilon \right)}^{N}(1-z)\right)}^{-k_{0}}\right]. \end{array}$$

We stress at this stage, we have not proposed a specific form for the distribution of rounds attended, π, and in this section present results for a general distribution.

### 2.2. Mean and parasite aggregation within the host population post-mass drug administration

We can use the probability generating function (2.4) to calculate the first and second moments for a given distribution π. The mean is

2.5 $$\begin{array}{c} m_{\mathrm{post}}=G^{\prime }{}_{X_{\mathrm{post}}}(1)=m_{0}G_{N}(1-\varepsilon ) \end{array},$$

and the variance is

2.6 $$\begin{array}{rl} \sigma_{\mathrm{post}}^{2} & ={G^{\prime \prime }}_{X_{\mathrm{post}}}(1)+{G^{\prime }}_{X_{\mathrm{post}}}(1)+{G^{\prime }}_{X_{\mathrm{post}}}{\left(1\right)}^{2}\\ & \begin{array}{c} =m_{0}{}^{2}\left(\frac{1+k_{0}}{k_{0}}\right)G_{N}({\left(1-\varepsilon \right)}^{2})+m_{0}G_{N}(1-\varepsilon )(1-m_{0}G_{N}(1-\varepsilon )). \end{array} \end{array}$$

There are many ways to characterize the aggregation for a given distribution of burdens. Some authors characterize aggregation using the dispersion index (also called the variance-to-mean ratio) [3,13]. In this analysis, we define aggregation of a distribution to be the following function of the mean, *m*, and variance, *σ*^2^,

2.7 $$\begin{array}{c} k:=\frac{m^{2}}{\sigma^{2}-m} \end{array},$$

which ensures the definition is consistent with the aggregation parameter defined for the negative binomial probability distribution. This means the aggregation of the post MDA distribution, *k*_post_, is

2.8 $$\begin{array}{c} k_{\mathrm{post}}:=\frac{k_{0}G_{N}(1-\varepsilon )}{\left(1+k_{0})G_{N}({\left(1-\varepsilon \right)}^{2})-k_{0}G_{N}^{2}(1-\varepsilon \right)}. \end{array}$$

In the full drug efficacy limit *ɛ* → 1

2.9 $$\begin{array}{c} k_{\mathrm{post}}=\frac{k_{0}m_{\mathrm{post}}}{(1+k_{0})m_{0}-k_{0}m_{\mathrm{post}}}. \end{array}$$

We can conclude from this that when the drug efficacy is high, the precise form of the compliance model has limited effect on the relationship between the mean and aggregation of the post chemotherapy distribution, and that when *m*_post_ is small the relationship is approximately linear with gradient *k*_0_/(*m*_0_*k*_0_ + *m*_0_).

## 3. Modelling systematic non-compliance

The aim of repeated MDA is to supply treatment to as large a proportion of the population as possible, ideally more than once, to suppress the prevalence of parasite infection and the mean intensity of infection to below a point (ideally reducing the effective reproduction number *R*_e_ < 1) at which a rapid resurgence of transmission and concomitant infection is unlikely to occur. The coverage for a round of chemotherapy is defined as the fraction of people who receive treatment out of the eligible population. It is practically very difficult to achieve perfect coverage on each round, and on average only a fraction *c_i_* ∈ (0, 1) receive treatment on the *i*th round (*i* = 1, … , *n*). If each host is equally likely to receive treatment in the *i*th round, then c_i_ also represents the probability of a randomly selected individual receiving treatment. We label this behaviour as full random non-compliance (and the converse—random compliance). In real MDA interventions, it is far more common to observe patterns of compliance where each individual does not appear to have the same probability of attendance [10,11]. For example, we may observe that individuals either attend all rounds, or no rounds, more frequently than would be expected if the probability of attendance is equal across the population. We label the behaviour where individuals attend either all possible rounds or none as full systematic non-compliance. In general, observed patterns, where there is variability in host behaviour, is better approximated by patterns where the probability of an individual attending in round *i*, *p*_i_, is sampled from a distribution of probabilities of attendance *f_i_* [10,11]. Note that in the fully random compliance model, we may consider the distribution to be the generalized distribution *f_i_*(*p*) = *δ*(*p* − *c_i_*) where *δ* is the Dirac-delta function, while in the fully systematic non-compliance model, the generalized distribution is *f_i_*(*p*) = *c_i_δ*(1 − *p*) + (1 − *c_i_*)*δ*(*p*).

The probability of an individual attending *N* out of a possible *n* rounds is given by the sum of independent Bernoulli trials with probabilities *p*_1_, … , *p*_n_. In particular, if each round has the same expected coverage, *c_i_* = *c* for each round, then *N* ∼ Binomial(*n*, *p*), where *p* is drawn from a distribution f with mean c. Given *N* is conditionally a binomial, a convenient choice for *f* is the beta distribution, as used by Dyson *et al*. since the beta distribution is the binomial distribution's conjugate prior [14]. The resulting distribution for *N* is the beta-binomial distribution with two parameters (*α*, *β*). To ensure that the expected coverage in each round is *c*, and the correlation of compliance with treatment between any two rounds is *ρ* ∈ (0, 1), the parameters of the beta-binomial distribution are uniquely specified as

3.1 $$\begin{array}{c} \alpha =\frac{c(1-\rho )}{\rho } \end{array}$$

and

3.2 $$\begin{array}{c} \beta =\frac{\left(1-c)(1-\rho \right)}{\rho }. \end{array}$$

**Figure 1.**
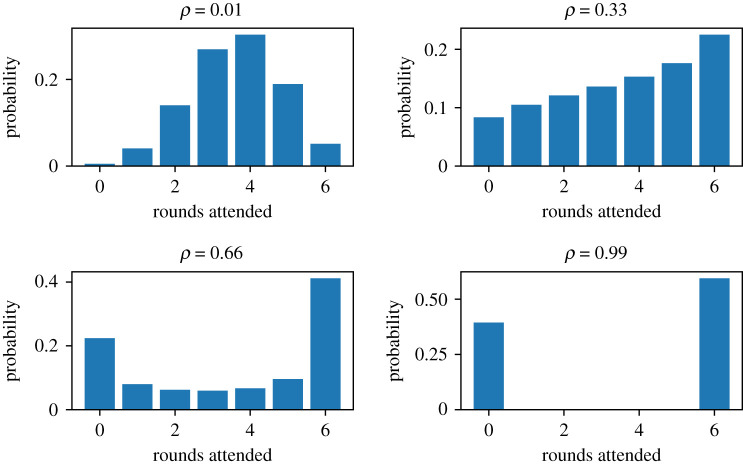
The beta-binomial probability distribution for rounds attended *N*, with number of MDA rounds *n* = 6, and coverage *c* = 0.6.

The correlation parameter *ρ* controls the strength of the systematic compliance. In the limit *ρ* → 0, the fully random non-compliance model is recovered, resulting in a binomial distribution for *N*. In the limit *ρ* → 1, the fully systematic non-compliance model is recovered, where hosts either attend all or no rounds. Figure 1 shows the distribution of *N* for various values of *ρ*.

With this beta-binomial distribution, the probability generating function for *N* is

3.3 $$\begin{array}{c} G_{N}(z)=\frac{2F1\;(-n,\alpha ,-\beta -n+1;z)}{2F1\;(-n,\alpha ,-\beta -n+1;1)} \end{array},$$

where _2_*F*_1_ is the hypergeometric function.

**Figure 2.**
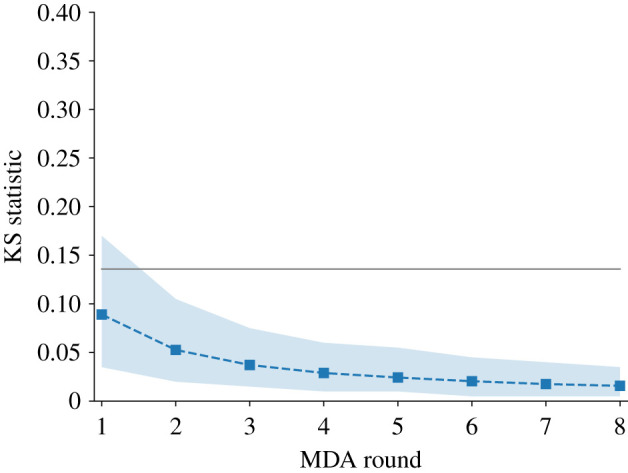
The two sample Kolmogorov–Smirnov test statistics for 200 random samples from the post-MDA distribution with parameters *n* = 8, *k*_0_ = 0.5, *ρ* = 0.25, *c* = 0.6, and a negative binomial with the same mean and aggregation. Blue markers show mean of 1000 trials; the shaded region indicates 95% of the distribution; the black line indicates the significance threshold at *α*_KS_ = 0.05.

In figure 2, we compare a post-MDA distribution to a negative binomial distribution with the same mean and value of the aggregation parameter *k*, by generating 200 samples from each and calculating the two-sample Kolmogorov–Smirnov (KS) test statistic. We find that the post-chemotherapy distribution is well approximated by a negative binomial, as indicated by KS test statistics that are below the *α*_KS_ = 0.05 significance level on each chemotherapy round.

We note that where good data are available, it may be possible to empirically fit models that generate the joint distribution for the probabilities of attending in each round, (*p*_1_, … , *p_n_*), where the random attendance probabilities *p*_i_ and *p*_j_ are not independent for *i* not equal to *j*, such as in a paper under review by Hardwick *et al.* which uses a Markov chain model. Such models with a greater number of free parameters will be able to better capture observed patterns of individual compliance. However, we suspect that they will not create a large difference in the distribution for the number of rounds attended, *N*.

## 4. Comparison with a stochastic nonlinear parasite transmission and treatment model

### 4.1. A stochastic nonlinear parasite model

In this section, we compare the mean and aggregation of the post-chemotherapy distribution to estimates that we obtain from a stochastic nonlinear dynamic model. First, we describe a general parasite model, in which hosts can be infected by parasites from a reservoir of infective eggs and larvae, which sexually reproduce within the host when mature. We let *X_t,i_* (superscript M) and *X_t,i_* (superscript *F*) be stochastic processes that represents the burden of males and female parasites in individual host within a population of size *n_h_*, indexed by time *t*, and host *i*, and let *L_t_* be the reservoir of infectious material at time *t*. The simple model is

4.1 $$\lambda_{i}∼\mathrm{Gamma}\left(\frac{\lambda }{k},k\right)\;\mathrm{initialized}\;\mathrm{at}\;t\;=\;0,$$

4.2 $$X_{t,i}^{M/F}\to X_{t,i}^{M/F}+1\;\mathrm{at}\;\mathrm{rate}\;0.5\;\lambda_{i}L_{t},$$

4.3 $$X_{t,i}^{M/F}\to X_{t,i}^{M/F}-1\;\mathrm{at}\;\mathrm{rate}\;\mu \;X_{t,i}^{M/F},$$

4.4 $$\mathrm{and}\;\;\;L_{t}=\underset{i}{\sum }F(X_{t,i}^{M},X_{t,i}^{F}),$$

where *λ*_i_ is the contact rate between host and infectious reservoir, *k* is the aggregation parameter, *µ* is the death rate of parasites within host. The function *F* describes the relationship between parasite burden and contribution to the reservoir *L_t_*. If we assume polygamous parasite mating (which is more suited to STH and filarial helminth species), where the number of mated parasite pairs in a host is zero if no male parasites are present and equal to the number of female worms otherwise, and a negative exponential density-dependent adult female fecundity function (see deterministic models for the outline form of the model [5,6]) then *F* is

4.5 $$\begin{array}{c} F(X_{t,i}^{M},X_{t,i}^{F})=X_{t,i}^{F}(X_{t,i}^{M}>0)\exp(-\gamma (X_{t,i}^{M}+X_{t,i}^{F}-1)) \end{array},$$

where the parameter *γ* controls the strength of density dependent fecundity.

**Figure 3.**
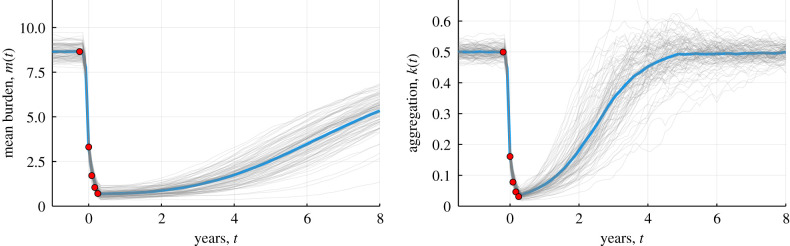
Mean burden (left) and aggregation (right) from 100 realizations of the nonlinear stochastic model (individual runs, grey, mean of runs, blue), with four rounds of monthly MDA and predictions from post-MDA distribution (red makers). Parameters: *k*_∞_ = 0.5, *µ* = 1/2.5 years^−1^, γ=0.08, *λ* = 3.2 (*R*_0_ = 0.5 *λ*/*µ* = 4.0). Population is seeded with an initial burden and allowed to move to an equilibrium of stable endemic infection. MDA is performed with coverage *c* = 0.65, non-compliance correlation *ρ* = 0.25 and drug efficacy *ɛ* = 0.95. The first round is conducted at *t* = 0 (years) and then every month for four months.

In figure 3, we plot the mean dynamics of the worm burden and prevalence (blue line, individual realizations in light grey), during four rounds of monthly MDA, and overlay the equivalent post-MDA distribution (red markers) which uses the assumption that there are no dynamics between MDA rounds. We see that the post-MDA distribution very accurately approximates the mean burden and aggregation through the rounds of MDA when the time between rounds is small. Because the relaxation of the aggregation *k*(*t*) is fast, the dynamics of the aggregation between rounds needs to be considered to obtain an accurate approximation of the aggregation if the time between rounds is large, so in the following section, we derive a linear model of the dynamics. Note that the simulated patterns are similar to those reported in Werkman *et al*. using an individual based stochastic model of parasite transmission [9].

### 4.2. A linear approximation to the dynamics

To approximate the dynamic relationship between the mean burden *m*(*t*) = E [*X_t_*] and aggregation *k*(*t*) = *m*(*t*)^2^/(*σ*(*t*)^2^ − *m*(*t*)), where *X_t_* = *X*^M^ + *X*^F^ and *σ*^2^ = var[*X_t_*], we fix the reservoir *L_t_* = L* which removes the nonlinearity from the model, and also the dynamical dependence between individuals in the same population. The linearized model is

4.6 $$\lambda^{*}∼\mathrm{Gamma}\left(\frac{\lambda }{k_{\infty }},k_{\infty }\right),$$

4.7 $$X_{0}=x_{0},$$

4.8 $$X_{t}^{}\to X_{t}^{}+1\;\mathrm{at}\;\mathrm{rate}\;\lambda^{*}L^{*},$$

4.9 $$\mathrm{and}\;\;\;X_{t}^{}\to X_{t}^{}-1\;\mathrm{at}\;\mathrm{rate}\;\mu \;X_{t}^{}.$$

Conditioned on $\lambda^{*}$, this model is an *M*/*M*/∞ queue (a well-studied stochastic model) and so conditionally the moments have the unique solution

4.10 $$\begin{array}{c} m(t)=\frac{\lambda^{*}L^{*}}{\mu }(1-e^{-\mu t})+x_{0}e^{-\mu t} \end{array}$$

and

4.11 $$\begin{array}{c} \sigma^{2}(t)=(1-e^{-\mu t})\left(\frac{\lambda L^{*}}{\mu }+x_{0}e^{-\mu t}\right). \end{array}$$

Using the laws of total expectation and variance to remove the conditioning on $\lambda^{*}$, the moments are

4.12 $$\begin{array}{c} m(t)=m_{\infty }(1-e^{-\mu t})+x_{0}e^{-\mu t} \end{array}$$

and

4.13 $$\begin{array}{c} \sigma^{2}(t)=\frac{m_{\infty }^{2}}{k_{\infty }}{\left(1-e^{-\mu t}\right)}^{2}+(m_{\infty }+x_{0}e^{-\mu t})(1-e^{-\mu t}) \end{array},$$

where *m*_∞_ = L*/*µ*. In the limit *t* → ∞, we have *m*(*t*) → *m*_∞_, *σ*^2^(*t*) → *m*^2^/*k*_∞_ + *m*_∞_, and *k*(*t*) → *k*_∞_. Further to this, since the limiting distribution of the *M*/*M*/∞ queue is Poisson, the limiting distribution of *X_t_* is negative binomial.

Next, we suppose that *X*_0_ has a negative binomial initial condition, with mean m_0_ and aggregation *k*_0_, (the common assumption for distributions of parasite burdens at baseline). Using the law of total expectation and variance, the moments are

4.14 $$\begin{array}{c} m(t)=m_{\infty }(1-e^{-\mu t})+m_{0}e^{-\mu t} \end{array}$$

and

4.15 $$\begin{array}{c} \sigma^{2}(t)=\sigma_{0}^{2}e^{-2\mu t}+\frac{m_{\infty }^{2}}{k_{\infty }}{\left(1-e^{-\mu t}\right)}^{2}+(m_{\infty }+m_{0}e^{-\mu t})(1-e^{-\mu t}) \end{array},$$

and hence the aggregation is

4.16 $$\begin{array}{c} k(t)=\frac{{\left(m_{\infty }(1-e^{-\mu t})+m_{0}e^{-\mu t}\right)}^{2}}{(m_{\infty }^{2}/k_{\infty })e^{-2\mu t}+(m_{\infty }^{2}/k_{\infty }){\left(1-e^{-\mu t}\right)}^{2}}. \end{array}$$

By eliminating *e*^−*µt*^ out of the above expressions, we find the following expression for the aggregation *k*(*t*) purely in terms of *m*(*t*) and the other parameters

4.17 $$\begin{array}{c} k(t)=\frac{m{\left(t\right)}^{2}(m_{\infty }-m_{0})}{(m_{\infty }^{2}/k_{\infty }){\left(m(t)-m_{0}\right)}^{2}+(m_{0}^{2}/k_{0}){\left(m(t)-m_{\infty }\right)}^{2}} \end{array}.$$

## 5. The effect of aggregation on the unstable equilibrium

### 5.1. Mean field approximation to the nonlinear stochastic process

By assuming that the distribution of macroparasites is distributed as a negative binomial with dynamically varying mean burden *m*(*t*) and constant aggregation *k*, Anderson & May [5,6] derived the mean field equation for the mean burden

5.1 $$\begin{array}{c} \frac{dm}{dt}=\mu (R_{0}\phi (m;k,z)f(m;k,z)-1)m \end{array},$$

where *R*_0_ is the basic reproduction number for macroparasite transmission, which is defined as the average number of female offspring that survive to reproductive age, produced by a single female macroparasite over the course of its lifespan in the absence of density dependent effects. For helminth models, this is a product of the rate egg of production per female helminth, the life expectancy of adult helminths, the life expectancy of infective larvae, and on the probability an infective larvae makes contact with an infective host. The function f(m;k,z) represents the negative density dependence caused by overcrowding of helminths within the host, which reduces egg output. When the density dependence of egg production within the host is exponential, with gradient of exponential decay γ, the function f has the form

5.2 $$\begin{array}{c} \;f(m;k,z)=\;{\left(1+\frac{m}{k}(1-z)\right)}^{-k-1} \end{array},$$

where z=exp(−γ). The mating function ϕ(m;k,z) represents the fraction of egg output that is fertilized. STH parasite species are generally assumed to be polygamous, which produces the mating function

5.3 $$\begin{array}{c} \phi (m;k,z)=1-{\left(\frac{1+((1-z)m/k)}{1+((2-z)m/2k)}\right)}^{k+1}. \end{array}$$

Schistosome species are generally assumed to be monogamous, and a result of this is that the density dependence effects does not neatly factorize into f(m;k,z) ϕ(m;k,z). However, the approximation

5.4 $$\begin{array}{c} \phi^{\prime }(m;k)=1-\frac{{\left(1-\omega \right)}^{k+1}}{2\pi }\int_{0}^{2\pi }\frac{1-\cos\theta }{{\left(1+\omega \cos\theta \right)}^{k+1}}d\theta , \end{array}$$

where ω=m/(m+k) which is found in the weak density dependence limit z→1, is considered to provide close approximation for the monogamous worm mating function [15].

**Figure 4.**
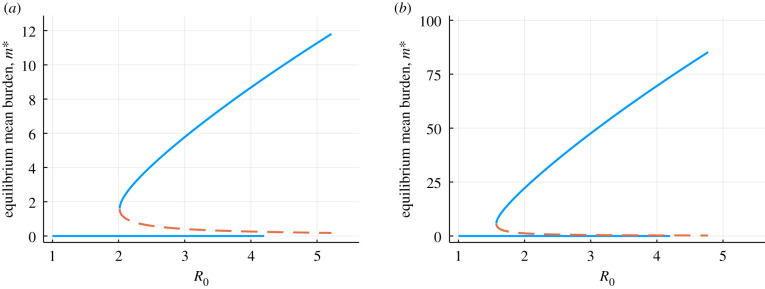
Saddle-node bifurcation of the ODE (30) with (*a*) polygamous mating function and typical hookworm parameters (*z* = 0.92, *k* = 0.5 [1,16]), and (*b*) monogamous mating function and typical *Schistosoma mansoni* parameters (*z* = 0.99, *k* = 0.5 [1,17]). Solid blue lines are the stable equilibria, orange dashed is the unstable equilibrium (breakpoint).

**Figure 5.**
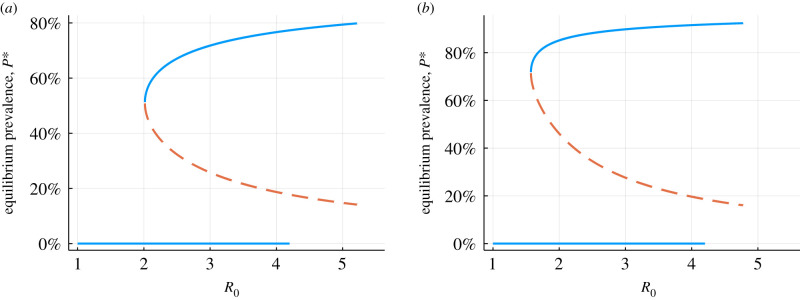
Saddle-node bifurcation of the ODE (30) with (*a*) polygamous mating function and typical hookworm parameters (*z* = 0.92, *k* = 0.5 [1,16]), and (*b*) monogamous mating function and typical *Schistosoma mansoni* parameters (*z* = 0.99, *k* = 0.5 [1,17]). Solid blue lines are the stable equilibria, orange dashed is the unstable equilibrium (breakpoint). Equilibrium prevalence is related to mean burden by *P* = 1 – (1 + *m*/*k*)^−*k*^.

The ODE (5.1) together with the density dependent effects ((5.1)–(5.4)) are well studied dynamical systems, and have a saddle-node bifurcation at $R_{0}=R^{*}>1$ . For $R_{0}<R^{*}$, there is one equilibrium (mean burdens *m*^∗^ for which the time derivative d*m*/d*t* = 0) at *m* = 0, which is stable. For $R_{0}>R^{*}$, there are two non-trivial equilibria (see figures 4 and 5). The density-dependent mating probability produces a critical value R* for parasite persistence is greater than one, because producing one fertile female offspring does not guarantee the presence of a male to mate with in the same host. Note, this definition of *R*_0_ is different to the more familiar microparasite definition of *R*_0_, which measures the average number of secondary infections of hosts produced by an index case in a wholly susceptible population

The existence of the unstable equilibrium, also referred to in the literature as the transmission breakpoint, is created by the requirement of both parasite sexes to be present in an individual host for mating and the production of fertile transmission stages, which produces an Allee effect in the system. If the mean burden is below the breakpoint there are not enough mated worm-pairs expected to be in the population to sustain transmission.

**Figure 6.**
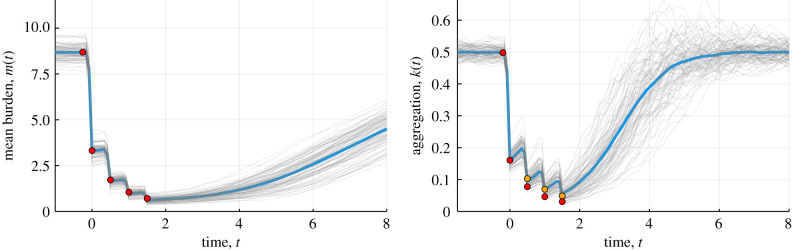
Mean burden (left) and aggregation (right) from 100 realizations of the nonlinear stochastic model (individual runs, grey; mean of runs, blue), with four rounds of six-monthly MDA and predictions from post-MDA distribution (red and yellow markers). Parameters: *k*_∞_ = 0.5, *µ* = 1/2.5 years^−1^, γ=0.08, *λ* = 3.2 (*R*_0_ = 0.5 *λ*/*μ* = 4.0). Population is seeded with an initial burden and equilibrated. MDA is performed with coverage *c* = 0.65, non-compliance correlation *ρ* = 0.25 and drug efficacy *ɛ* = 0.95. The first round is conducted at *t* = 0 (years) and then every six months for four rounds. Red markers are post-MDA predictions assuming no intra-MDA dynamics, yellow markers are the correction for the intra-MDA dynamics obtained from the linear approximation to the dynamics.

The aim of MDA is to reduce the mean burden to below the breakpoint (or more generally to reduce the value of the basic reproductive number below the level needed to sustain transmission), after which no further rounds of MDA are necessary for the mean burden to decay to zero. Crucially, the location of the breakpoint is dependent on the degree of parasite aggregation, inversely measured by k, which increases with more rounds of MDA, and as seen in figure 3 and figure 6. As shown in this paper, its value is also dependent on the coverage and level of compliance. That is, each round of MDA reduces the mean burden, but also reduces the value of k and lowers the breakpoint value towards zero. If (*m*^(*i*)^, *k*^(*i*)^) denotes the mean and aggregation after the *i*th round of MDA, then the number of rounds after which the disease can no longer sustain itself, *n*^∗^, is the smallest number of rounds for which the time gradient of *m*(t) is negative, that is

5.5 $$\begin{array}{c} n^{*}=\min\left\{i\in N:\frac{dm}{dt}=F(m^{\left(i\right)};k^{\left(i\right)})<0\right\}, \end{array}$$

For an initial mean parasite burden m_0_ and aggregation *k*_0_ we are able to calculate *n*^∗^ up to a maximum of 15, for given MDA coverage c, non-compliance correlation *ρ* and drug efficacy *ɛ*, using the analytical expressions for the mean worm burden and aggregation level from §2. It is unrealistic for a location to receive 15 rounds of continuous MDA, but we use this number to indicate that it would be practically impossible to reach the breakpoint with the parameters under consideration.

**Figure 7.**
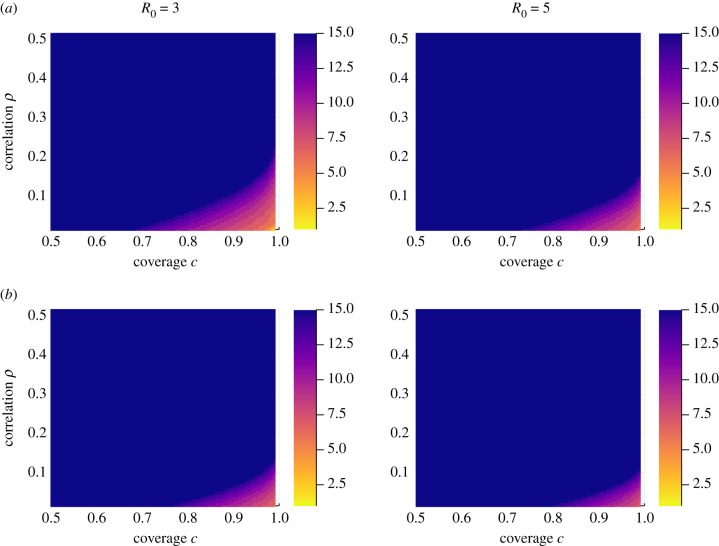
Number of rounds, *n*^∗^, to reach breakpoint for different coverages *c* and non-compliance correlation *ρ*, up to a maximum of 15 rounds. Parameters: row (*a*) polygamous mating function and typical hookworm parameters (*z* = 0.92, *k* = 0.5 [1,16]), row (*b*) monogamous mating function and typical *Schistosoma mansoni* parameters (*z* = 0.99, *k* = 0.5 [1,17]). Left column *R*_0_ = 3, right column *R*_0_ = 5. Drug efficacy *ɛ* = 0.95.

Figure 7 shows *n*^∗^ for coverages between 0.5 and 1 and compliance correlations between 0 and 0.5, for *R*_0_ = 3 (left column) and *R*_0_ = 5 (right column), and for a polygamous mating helminth with typical hookworm parameters (top row) and monogamous mating helminth with typical *Schistosoma mansoni* parameters (bottom row). For each parameter combination, increasing the coverage c decreases *n*^∗^ while increasing non-compliance correlation *ρ* increases *n*^∗^. The majority of the MDA parameter space explored has *n*^∗^ > 15, where it appears that either only a very large number of MDA rounds will result in decaying mean burdens post MDA cessation, or reaching the breakpoint is not possible with any number of rounds. This means that for such parameters, on average there are always high-risk individuals who have persistently missed treatment either from personal choice or failure to gain access to treatment, and as a consequence are able to cause a resurgence of the infection in the rest of the host population.

The region of coverage and correlation which separates areas where the breakpoint is achieved in under 10 MDA rounds, and those which require 15 or over is relatively narrow, which means that there will be a large amount of uncertainty regarding how many rounds are needed to reach a breakpoint if the MDA implementation parameters are in this region.

**Figure 8.**
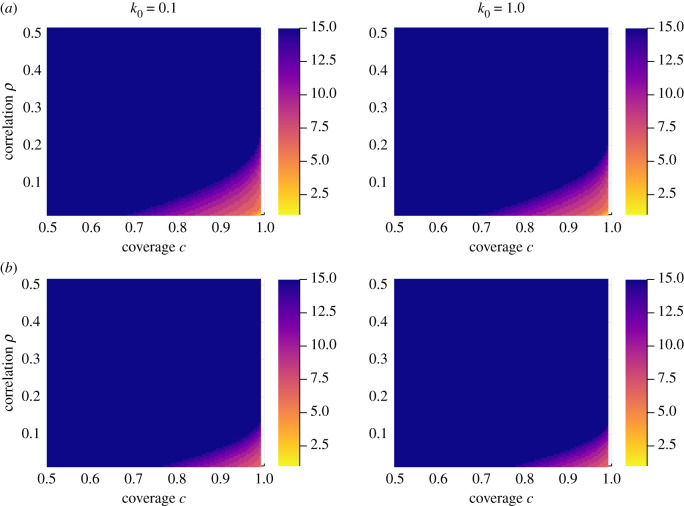
Number of rounds, *n*^∗^, to reach breakpoint for different coverages *c* and non-compliance correlation *ρ*, up to a maximum of 15 rounds. Parameters: row (*a*) polygamous mating function and typical hookworm parameters (*z* = 0.92, *R*_0_ = 3 [1,16]), row (*b*) monogamous mating function and typical *Schistosoma mansoni* parameters (*z* = 0.99, *R*_0_ = 3 [1,17]). Left column *k*_0_ = 0.1, right column *k*_0_ = 1. Drug efficacy *ɛ* = 0.95.

Figure 8 shows *n*^∗^ for different initial degrees of parasite aggregations *k*_0_. Published studies for helminth parasites put these values in the range of 0.1 to 1.0 [1]. *k*_0_ does not greatly affect the number of rounds required to reach the breakpoint.

## 6. Discussion

The definition of a transmission breakpoint in either the mean worm burden or prevalence (by a very sensitive diagnostic) of helminth infections of humans, below which parasite transmission although continuing is too low to sustain the persistence of infection in the longer term, is central to studies of the epidemiology and control of infectious diseases [1]. For microparasites, the boundary *R*_0_ = 1 provides the definition of this breakpoint. For helminth infections, the definition is more complex, and often confusing to those not familiar with mathematical models of parasite transmission. Because of the influence of the need for sexual reproduction to sustain transmission involving the presence of both female and male worms within the human host, the breakpoint may lie at a point where *R*_0_ > 1. The exact location of the breakpoint is determined by the degree of parasite aggregation within the host population since highly aggregated patterns even when overall prevalence is low, ensure male and female worms exist together in the same host. The greater the degree of parasite aggregation the lower the breakpoint is in terms of either the mean worm load or the prevalence of infection and in the limit where all parasites are within one host (the negative binomial *k* tends to zero), the breakpoint is convergent on a mean burden or prevalence of zero. This has been known for some time [6].

What has not been appreciated, however, is that repeated rounds of MDA, the favoured control option for human helminth infections, tend to drive parasite aggregation to higher and higher levels (very small *k* values) [9]. Very recent studies point to persistent non-adherence to treatment being the causative mechanism [10,11]. It is too early to say if this is universally true for all helminths, and all control programmes, since very few studies of scale have been completed to date on individual compliance to treatment over many rounds of MDA. Those that have been done recently are focused on STH. More needs to be done for the filarial worms and schistosome infections.

In this paper, we build a probabilistic framework for a stochastic individual based model of transmission and control that permit assessing the influence of non-compliance in MDA rounds on the change in the distribution of parasites over time. We also make some simple approximations to obtain some analytical insights into where the breakpoint in transmission is influenced by parasite aggregation. The results of these analytical and numerical studies show clearly that non-compliance in a small fraction of the treated population first drives aggregation higher (*k* smaller) and concomitantly makes transmission elimination more difficult than predicted by deterministic models and stochastic models that assume random compliance at each round of MDA [18]. This implies that in practice transmission elimination will be more difficult and require both higher levels of coverage in MDA rounds across all age groups and an increased focus on what are the causes of non-compliance by the few and how can they be remedied.

The analytical and numerical results reported in this paper may be too pessimistic because of mean field approximations to what are stochastic fluctuations that become highly important when population sizes of parasites (at very low prevalences) become very small. The mean field approximation is only robust for large and well-mixed populations. For small populations, random fluctuations in both parasite population dynamic processes (successful transmission events, meeting a mate and deaths) and MDA delivery to individuals are likely to cause interruptions in effective transmission, regardless of the mean expected behaviour. Other simplifications that may impact the pessimistic conclusions are the approximations made such as no dynamics between rounds of treatment, and neglecting age structure which is important for the transmission and control of disease that primarily effect children such as schistosomiasis. As such, the results from this analysis should not be used as precise predictions for how many rounds are needed to reach a transmission breakpoint for specific scenarios.

The major practical lesson emerging from these analyses is that much more attention must be paid to both measuring individual compliance to treatment and to what causes non-compliance with the aim of ensuring all those that it is aimed to treat get treated [19,20]. Finally, this study also highlights the importance of sustainable surveillance systems for monitoring the real-time distributions of helminths in the target communities, because high coverage and compliance is particularly important when the distribution is highly aggregated.

## Data accessibility

All data used in this study are generated by numerical evaluation of the equations and output of stochastic models. All numerical and plotting code are available upon request.
